# State-of-the-art reviews predictive modeling in adult spinal deformity: applications of advanced analytics

**DOI:** 10.1007/s43390-021-00360-0

**Published:** 2021-05-18

**Authors:** Rushikesh S. Joshi, Darryl Lau, Justin K. Scheer, Miquel Serra-Burriel, Alba Vila-Casademunt, Shay Bess, Justin S. Smith, Ferran Pellise, Christopher P. Ames

**Affiliations:** 1 Department of Neurological Surgery, University of California, San Francisco, 400 Parnassus Avenue, A850, San Francisco, CA 94143 USA; 2 Center for Research in Health and Economics, Universitat Pompeu Fabra, Barcelona, Spain; 3 Vall d’Hebron Institute of Research (VHIR), Barcelona, Spain; 4 Denver International Spine Center, Presbyterian St. Luke’s/Rocky Mountain Hospital for Children, Denver, CO USA; 5 Department of Neurosurgery, University of Virginia Medical Center, Charlottesville, VA USA; 6 Spine Surgery Unit, Hospital Vall d’Hebron, Barcelona, Spain

**Keywords:** Spinal deformity, Artificial intelligence, Machine learning, Technology, Predictive model

## Abstract

Adult spinal deformity (ASD) is a complex and heterogeneous disease that can severely impact patients’ lives. While it is clear that surgical correction can achieve significant improvement of spinopelvic parameters and quality of life measures in adults with spinal deformity, there remains a high risk of complication associated with surgical approaches to adult deformity. Over the past decade, utilization of surgical correction for ASD has increased dramatically as deformity correction techniques have become more refined and widely adopted. Along with this increase in surgical utilization, there has been a massive undertaking by spine surgeons to develop more robust models to predict postoperative outcomes in an effort to mitigate the relatively high complication rates. A large part of this revolution within spine surgery has been the gradual adoption of predictive analytics harnessing artificial intelligence through the use of machine learning algorithms. The development of predictive models to accurately prognosticate patient outcomes following ASD surgery represents a dramatic improvement over prior statistical models which are better suited for finding associations between variables than for their predictive utility. Machine learning models, which offer the ability to make more accurate and reproducible predictions, provide surgeons with a wide array of practical applications from augmenting clinical decision making to more wide-spread public health implications. The inclusion of these advanced computational techniques in spine practices will be paramount for improving the care of patients, by empowering both patients and surgeons to more specifically tailor clinical decisions to address individual health profiles and needs.

## Introduction

Over the past couple decades, our knowledge of adult spinal deformity (ASD) as a complex disease has increased immensely. It is now well established that ASD is a heterogeneous entity that can cause significant pain and disability in patients, with worse deformity exacerbating these symptoms [1–4]. As our understanding of ASD as a complex disease has grown, so has the body of literature describing surgical management of this condition—resulting in a surge in popularity and wide-spread utilization of these surgical techniques. Studies have shown with a high degree of reproducibility that surgical intervention can achieve significant correction of spinopelvic parameters and dramatically improve various health-related quality of life (HRQOL) measures in patients, especially those who are severely disabled [5–18]. Despite the potential benefits of surgical management, these techniques are invasive, often requiring significant bony resection through osteotomies as well as soft tissue release to obtain the desired results [4, 19]. While powerful in their ability to correct pathological spinal alignment, surgical approaches to deformity correction are also associated with relatively high risk for both perioperative and long-term complications, and present a significant impact on healthcare systems through direct cost [13, 15, 16, 18, 20–23].

Due to the extensive variability of ASD presentation and the many factors pertinent to patients’ outcomes, ASD offers a unique opportunity for the application of advanced analytics in spine surgery. Throughout the history of operative and nonoperative management for ASD, spine surgeons have relied on their clinical judgment and large, retrospective studies to better inform decision making and to counsel patients regarding their treatment plan. Often times, the personal experience of surgeons provided the information, and this was heavily dependent on the surgical volume and exposure to ASD cases at various spine centers. While relatively rare in the spine literature, early predictive models helped us decipher some of the subtleties of spine surgery outcomes, with even fewer focusing on surgical correction of ASD and its associated risks and outcomes [24–29]. However, these efforts relied primarily on the application of statistical models such as linear/logistic regressions to identify pertinent information. While useful to identify ‘predictors’ of specific outcomes, the outputs of odds ratios and relative risk generated by regression models are difficult to interpret for both patients and physicians. The utility of regression models lies in their ability to identify the relationships and associations between variables, and thus make inferences about a generalized population. While statistical models can also be used for predictive purposes, this is not their strength, and the generalizations made at the population level have minimal applicability for the intricacies of patient-specific interactions. The primary purpose of machine learning models on the other hand, is to make accurate and repeatable predictions for new data based on patterns learned from old data.

As we transition into an era of medicine largely influenced by the digitization of data through the incorporation of electronic medical records, we have gained access to an astounding amount of patient information that can be used to create more robust and complex analytics. In conjunction with this explosive growth in available medical data, our ability to process this information through refined computational algorithms has progressed as well. Over the past few years alone, we have seen various disciplines within medicine gradually adopt artificial intelligence (AI) techniques primarily through the use of machine learning methods to process and analyze unprecedented amounts of data. Within neurosurgery specifically, several groups have taken significant strides towards implementing artificial intelligence into clinical practice. Titano et al. showed at a prominent academic center in New York that a machine learning framework utilizing a 3D convolutional neural network (CNN) could successfully triage radiology studies to help monitor for acute neurologic events [30]. The algorithm augmented human performance by prioritizing the radiology workflow and dramatically reduced processing and interpretation times for alerting physicians. Similarly, a group at Michigan devised a tool to facilitate intraoperative tissue diagnosis for tumor surgery using stimulated Raman histology (SRH) and CNNs [31]. Their integrated system allows for prediction of diagnosis in near real-time at the bedside, as well as identification of tumor-infiltrated regions for examining margins during surgery. The elegance of machine learning models is illustrated by their ability to implement complex mathematical models to identify patterns and relationships between perceived heterogeneous and unrelated data, and then to use these patterns to make highly accurate predictions for newly available data. More recently, spine surgeons have pioneered the incorporation of these analytics for myriad applications ranging from predicting outcomes to cost analysis, and in this review, we will discuss several of these advances in addition to highlighting the immense potential of machine learning for future studies.

### Trends in ASD surgery in the last decade

Given the prevalence of surgical utilization for ASD in the global spine community, it is imperative to first understand the true impact of this disease. As the number of ASD surgical cases continues to increase exponentially with respect to total volume as well as rate per 100,000 adults, there is concern among both physicians and healthcare payers regarding the reported rates of complications and the burgeoning cost of treatment [32]. To better understand this information, the European Spine Study Group (ESSG) and International Spine Study Group (ISSG) conducted a review of prospectively collected data spanning over 2000 patients operated on from 2010 to 2016 to better characterize global trends in ASD surgery. Through an international collaboration of five countries (and two continents) encompassing numerous spine centers and over 50 surgeons, data encompassing demographic, surgical, radiological and HRQOL metrics such as the Oswestry Disability Index (ODI), and Short Form-36 (SF-36) and Scoliosis Research Society-22 (SRS-22) health surveys was obtained. All patients included in the combined prospective database had greater than two years of follow-up data, with metrics collected at 3, 6, 12, and 24 months postoperatively. This combined ISSG-ESSG database represents the best available information regarding surgical outcomes for ASD.

**Fig. 1:**
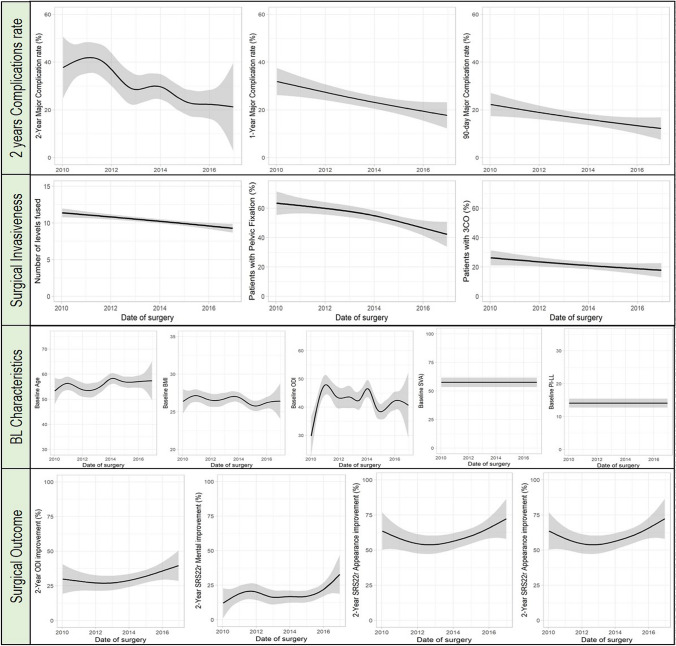
Trends from 2010 to 2016 in ASD surgery. From top to bottom: 2-year complication rates, measures of surgical invasiveness (including # of levels fused, % with pelvic fixation, and % undergoing 3CO), baseline characteristics of ASD surgical candidates, and surgical outcomes (measured by ODI and SRS-22 score improvements)

Of the 2286 patients included in the combined dataset, a total of 1151 patients operated on at 17 different sites met inclusion criteria. While baseline characteristics of patients including age, HRQOL scores, sagittal imbalance and ASA grade did not change from 2010 to 2016, there was a significant increase in overall patient recruitment (OR: 1.64, *p* < 0.01). In addition to the large increase in new patients undergoing ASD surgery, there was a sustained reduction in both major and minor complications observed at 90 days (major OR: 0.54, minor OR: 0.48; *p* < 0.01 for both), one year (major OR: 0.59, minor OR: 0.59; *p* < 0. 01) and two years of follow-up (major OR: 0.55, minor OR: 0.66, *p* < 0.01). Along with the reduction in complication rates observed over the past decade, the combined dataset also demonstrated a significant decrease in two-year reintervention rate (OR: 0.51, *p* < 0.01) as well as surgical invasiveness as defined by number of fused segments (OR: 0.81, *p* < 0.01), patients undergoing pelvic fixation (OR: 0.66, *p* < 0.01), and patients undergoing three-column osteotomies (3CO) (OR: 063, *p* < 0.01). It is important to consider that these trends pertain specifically to high-volume spine deformity centers, and that decreasing invasiveness along with less pelvic fixation and osteotomies is a concurrent observation, rather than a causative relationship due to increased ASD literature and surgeon experience. Notably, this decrease in patient morbidity was also accompanied by an improvement in patient HRQOL scores (ODI: 26% in 2010 vs. 40% in 2016, *p* = 0.02 and SRS-22 OR: 1.16, *p* = 0.13) in addition to degree of sagittal correction as measured by pelvic incidence-lumbar lordosis (PI-LL) mismatch (OR: 1.11, *p* = 0.19) [33]. In summary, it is clear that as surgeons have refined techniques for surgical correction of ASD over the last decade, there has been a marked decrease in complications and reoperations, while quality of life gain in patients has improved (Fig. 1). The ISSG and ESSG databases also underscore the significance of mutually compatible, large, prospective datasets containing high-quality data, which is of paramount importance when considering the implementation of advanced analytics.

## Understanding the methodology behind predictive modeling

**Fig. 2:**
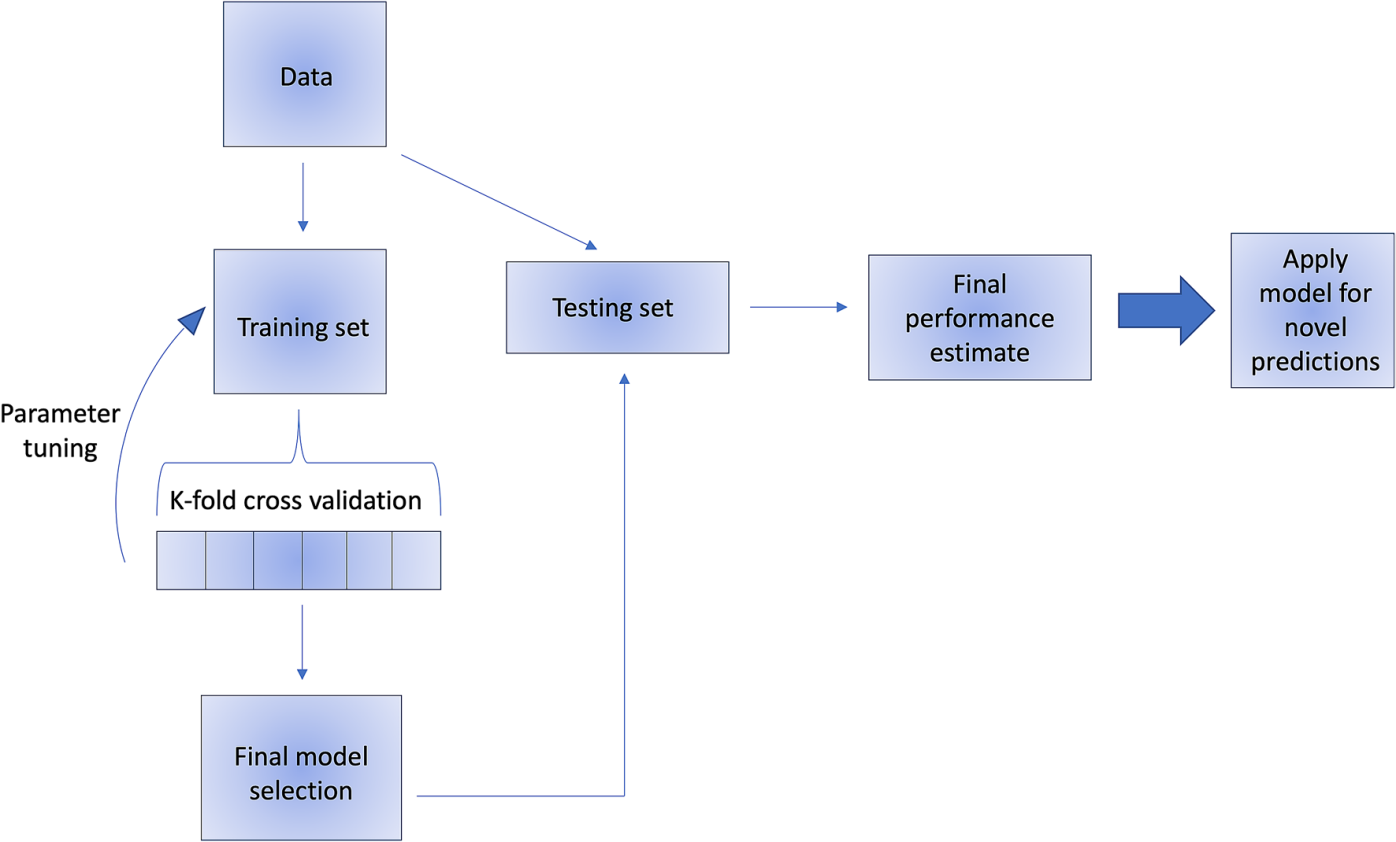
Flow chart demonstrating the general process of training, validating, and testing utilized during the development of machine learning models. This diagram shows how training/test datasets are generated from the original data (usually 80/20 split). The training data is then split again (generally 80/20) into a training set and validation set, most often using a technique called cross-validation. The training data is randomly split 80/20k-number of times, such that the model learns from the training set, and then parameter tuning is done with the validation set k-number of times; ultimately the learned models are averaged to select the optimal one. The resulting model is then tested on a distinct test set for final performance evaluation, usually given by % accuracy and AUC values. The model can then be deployed to make predictions on new data

To effectively utilize machine learning for predictive modeling, it is critical to understand the concepts and methods behind their implementation. At is core, AI represents the creation of a system that mirrors our innate ability to process information and dynamically learn as we are exposed to new situations. As it attempts to recapitulate our natural intelligence, AI makes use of numerous computational techniques, most commonly machine learning, which is considered a subset of AI. One of the core principles of machine learning is the idea of “training” algorithms on data that is available, and allowing the algorithm to determine mathematical relationships between variables inherent in the data. By removing the process of manually coding or interrogating relationships between selected variables, machine learning eliminates user bias regarding which variables are relevant or not for the desired analysis—often relationships that are not intuitively apparent can be identified by these methods. Traditional statistics including linear/logistic regression is hypothesis driven, and as such relies on many assumptions that are often not generalizable. Hypothesis-generated studies inherently require selection of predictor variables, which limits factor inclusion and can lead to omitted variable bias due to possible confounders being missed. Conversely, machine learning allows for the wide-spread inclusion of input variables and relies on robust algorithms to determine correlative relationships within the data. The power of machine learning techniques becomes readily evident in the context of ASD surgery, where patients often embody highly variable symptoms and medical profiles. Once algorithms are trained on available data, they are then “tested” on separate test sets, to evaluate the accuracy and performance of the constructed model. The test set gives the user an idea of how well the model will perform when deployed prospectively on novel data. Generally, data acquired for predictive model generation is split 80:20 or 70:30 into training and testing sets, respectively. Model training itself then tends to follow an iterative process, in which various models are tested for efficacy using a technique called cross-validation. In cross-validation, the training data is repeatedly partitioned in a random manner, such that in each iteration a portion of the actual training data is cordoned off as a “validation set”, to serve a similar purpose to the test set and allow for parameter tuning and model optimization. A summary of this process is depicted in Fig. 2. Once model performance is deemed sufficient on the test set, it can then be prospectively applied to new data to make specific predictions and determinations.

### Statistical models vs. machine learning: strengths, limitations, and common misconceptions

While it is important to recognize that statistical models still serve a vital role in outcomes research, there are several distinguishing factors regarding their applications when compared to machine learning predictive models. First and foremost, statistical models exist to characterize the relationship between data and an outcome variable, allowing us to infer the relationships between variables and test different hypotheses. Machine learning on the other hand is a computationally intensive technique that derives its utility from being able to process extraordinarily large amounts of data spanning diverse and heterogenous variables to make highly accurate and repeatable predictions. What statistical models lack in predictive ability however, they make up for in ease of interpretability. Predictive models created by machine learning algorithms offer far more powerful predictive capabilities, but as a result are often more difficult to interpret given their complexity.

Despite their seemingly limitless potential, there are several components integral to the proper development of predictive models. One of the most essential requirements is having access to robust data. Having a large amount of data is in itself not sufficient for applying machine learning methods. It is essential that the data be high-quality as well, such that a smaller matrix of reliable and high-quality data will be more useful than a larger matrix of outdated or inaccurate data. The quality of the data can be reflected in its consistency (ensuring data is all labeled appropriately and consistently for given attributes), accuracy (numbers accurately reflect the given attribute without typos or mistaken entries), completeness (minimal missing values for attributes), and the absence of duplicate or corrupted data entries. Sparsely populated data or overly complex models can also result in a phenomenon termed “overfitting”, where a machine learning model specifically caters too closely to the data it was trained on, and as a result loses accuracy when being applied to novel data. These issues can be mitigated by acquiring larger or higher quality datasets, as well as with diligent optimization of model parameters and adherence to principles such as cross-validation and strict training/testing as described earlier. Other techniques to avoid overfitting include using ensemble methods, which are machine learning methods that combine predictions from several different models to optimize the overall predictive ability, and bootstrapping. Bootstrapping is a statistical technique that involves sampling with replacement from a dataset, to estimate parameters about the entire dataset/population. Bootstrapping can be performed over several iterations, and the benefit of sampling with replacement is that some data entries may be considered zero, once, or more times, and thus expected variance is lowered as each bootstrapping iteration will be independent from its peers. Ensemble methods include ‘bagging’ (also known as bootstrap aggregating), which involves training multiple, complex models in parallel using bootstrapped samples and then averaging the responses of each of the models, and ‘boosting’ which trains simpler models in sequence using the entire training set, such that each subsequent model builds upon and learns from the failures of its predecessor (i.e. misclassified values or incorrect predictions). In contrast, statistical models still allow the user to make generalizable inferences using relatively small amounts of data. While observational studies can suggest average outcomes from specific interventions across entire populations, it is impossible to conduct accurate comparisons between observed outcomes in a specific patient, and hypothetical outcomes that may have risen from alternative management strategies with simple statistical models. For ASD surgery in particular, this is where predictive models derived by machine learning can have a significant impact—given the large spectrum encompassed by ASD patients, the incorporation of machine learning algorithms into predictive analytics can offer unprecedented prognostic information to augment the decision making of surgeons and bolster their ability to counsel patients. This granularity can help tailor treatment regimens to a patients’ specific needs, helping deliver a more personalized form of healthcare. In this current era of widely accessible computational programs, it will be imperative that physicians and researchers keep in mind these fundamental principles when reviewing studies in the literature, and meticulously follow the appropriate steps of model generation to avoid sharing misleading results.

## Frailty as a predictor of surgical outcomes

To help quantify and stratify the significant heterogeneity in the clinical presentation of ASD patients before predictive analytics, metrics such as the ASD frailty index (ASD-FI) were developed [34]. The concept of frailty as a medical diagnosis is relatively novel, and originally came about as a result of trying to explain differences in chronological age and physiological age [35]. Frailty represents a decrease in an individual’s physiological function, and was devised to help predict mortality and independence in the nonoperatively treated elderly population [36, 37]. It was later shown to be a better predictor of perioperative outcomes than age alone, as the multisystem impairments present in patients with high degree of frailty result in diminished physiological response to surgery-related stressors [38–41]. By adapting the idea of patient frailty as a predictor of surgical outcomes to spine surgery, the ASD-FI provided deformity surgeons with a tool to comprehensively profile ASD surgical candidates as part of their preoperative evaluation. The ASD-FI was validated in multiple prospectively collected ASD datasets and proved to be an effective method of preoperative risk stratification, showing that greater patient frailty was associated with worse outcomes including greater risk of major complications, proximal junctional kyphosis, pseudarthrosis, deep wound infection, wound dehiscence, reoperation and prolonged hospital stay [34, 42, 43].

Following the successful inclusion of the ASD-FI for evaluating thoracolumbar deformity patients, similar methods were subsequently applied for cervical deformity. As a result, the cervical deformity (CD) frailty index (CD-FI) was developed and subsequently modified for ease of implementation as the modified CD-FI (mCD-FI) [44, 45]. Similar to its thoracolumbar predecessor, the CD-FI and mCD-FI were shown to correlate with increased length of stay (LOS), neck pain, decreased HRQOL and greater postoperative complication risk; thus, providing surgeons with a robust clinical tool for preoperative risk stratification in CD surgical candidates.

The adoption of metrics such as the frailty indices used for both ASD and CD represent a significant paradigm in the generation of predictive models. The utilization of frailty indices demonstrate how more traditional statistical methods can still be used to elucidate drivers of postoperative outcomes, and why hypothesis-driven studies still serve a critically important function in clinical studies. However, despite the important correlative analysis exhibited by the use of novel frailty indices, the final outcome of an odds ratio has limited applicability to individual patient cases, instead representing a general correlation across a broad population. The impetus behind these research efforts is to ultimately create better systems for prognosticating patient outcomes. By identifying an important correlative factor and its constituent features, the frailty index studies highlighted several important variables which can then be included in machine learning predictive models to better prognosticate patient outcomes. To do so will require synergy between the development of novel metrics such as the frailty indices to better characterize patient profiles, and rigorously constructed predictive models, that can utilize this information. This combination of statistical methods and machine learning algorithms will serve to enhance patient counseling during clinic visits and bolster the armamentarium of spine surgeons.

## Overview of predictive models for ASD surgery

### Early predictive models

To date, spine surgeons have already begun to make significant strides in the creation of more complex predictive models through the implementation of machine learning techniques. The most common methods currently being employed focus on the use of decision tree-based learning models. In general, these algorithms utilize the creation of classification or regression trees to predict a desired variable such as complication risk or a specific outcome. In generating these predictive models, a variety of variables are incorporated as input features for model training. These variables can include patient demographic information, comorbidities, comprehensive indices such as the Charlson Comoborbidity index (CCI) and FI, radiographic parameters, surgical characteristics, HRQOL scores and intraoperative information. Different techniques such as bootstrapping or ensemble methods have also been judiciously used to combine several different (and possibly weaker) algorithms into a single, stronger classifier, to minimize overfitting while offering improved predictive value. These predictive analytics have been widely applied across the spectrum of ASD surgery, including prediction of intraoperative [46], perioperative [47, 48] and postoperative complications and outcomes [49–56].

While most applications of predictive models focus on determination of postoperative outcomes, Durand et al. developed a predictive model for intra- and postoperative blood transfusion requirements with a cohort of 1,029 ASD patients. Using an 80:20 split for training and test sets, their final decision tree and random forest models predicted transfusion rates among ASD patients with area under the curve (AUCs) of 0.79 and 0.85, respectively [46]. The random forest model offered very good predictive capability as measured by its AUC (better than the single classification decision tree), with the most influential variables for predicting transfusion being operative duration, surgical invasiveness, hematocrit, weight and age. Separate models were also created by Safaee et al. and Scheer et al. to predict LOS [47], and major early complications in ASD [48], respectively. When assessing patient LOS, a generalized linear model was trained on bootstrapped data consisting of 653 patients and tested on an independent test set of 240 patients to yield a predictive accuracy of 75.4% within two days of actual reported values [47]. Top predictors of LOS identified by Safaee et al. included staged surgery, C7 sagittal vertical axis (SVA), number of posterior levels fused, and CCI. The utility of being able to predict a patient’s LOS lies in its potential to identify high-risk patients and aid in point-of-care decision making postoperatively. The model developed by Scheer et al. to predict major complications at the intraoperative stage and within 6 weeks postoperatively implemented an ensemble of decision trees using bootstrapped models to produce a model with an AUC of 0.89 [48]. A total of 20 variables were highlighted as important predictors of intraoperative and perioperative complications, with the top predictors including age, leg pain, ODI, number of decompression levels and number of interbody fusion levels, followed by several HRQOL metrics and radiographic parameters. While decision trees generally have weaker predictive ability than more complex algorithms like random forest models, their simplicity makes them easier to interpret and understand, and using bootstrapping and ensemble learning can reduce the risk of overfitting the training models.

Building on the success of the earlier described applications, predictive analytics have also been deployed for assessment of a variety of postoperative outcomes including proximal junctional failure (PJF) and proximal junctional kyphosis (PJK) [49, 50], pseudarthrosis [51], and major complications at 2-years [52]. Scheer et al. were one of the first groups to report the use of predictive analytics for detecting PJF or clinically significant PJK in their study utilizing decision trees and bootstrapped models in a cohort of 510 ASD patients. Their final model demonstrated an overall accuracy of 86% with AUC of 0.89, highlighting the feasibility of trying to predict PJF and PJK rates following corrective ASD surgery [49]. This study was subsequently followed-up by Yagi et al. who supplemented the model described by Scheer et al. by including bone mineral density score as one of the input variables—this addition produced a predictive model with 100% accuracy in the test set, albeit using a much smaller cohort of 145 patients [50]. To broaden the scope of these models, these two groups continued to delve further by developing tools for prognosticating pseudarthrosis and major complication rates at 2-year follow-up. Scheer et al. implemented similar ensemble decision tree methods combined with bootstrapped models, incorporating 21 variables from a total of 82 initially assessed, to generate a model with 91% accuracy and AUC of 0.94 to predict pseudarthrosis at 2-years [51]. Interestingly, the top predictors for PJF and PJK were markedly different from those of pseudarthrosis. Major predictors of PJF and clinically relevant PJK included age, lower instrumented vertebrae (LIV) and preoperative SVA, while the top three predictors for pseudarthrosis were the LIV, use of bone morphogenic protein (BMP) and the max coronal cobb angle. The beauty of machine learning is that these relationships between predictor variables and outcomes are intrinsically learned from the data, often revealing novel insights. Yagi et al. further generalized the 2-year pseudarthrosis predictive model to encompass any major complication at 2-years, and were able to achieve a test accuracy of 92% with AUC 0.96 in a cohort of 195 patients [52]. A few of these studies reporting very high accuracy and AUC metrics were conducted with small cohorts, and as such need to be carefully reviewed in this context as this can be a cause of overfitting due to the limited sample size of training data. Lastly, going beyond just complication risk, in a novel application, Passias et al. devised a predictive model to assess cervical malalignment following thoracolumbar ASD surgery. Their model predicted cervical malalignment with AUC of 0.89, and demonstrated that patients with increased C2-T3 cobb angle at baseline and higher numbers of Smith-Peterson osteotomies (SPOs) performed had significantly higher rates of poor cervical alignment following surgery [53]. While some of these studies make use of relatively smaller datasets as mentioned earlier, this only serves to highlight the importance of ensuring the input data is high-quality, and reiterates the need for multi-institutional and multi-national collaborative efforts to generate larger, prospectively collected databases for ASD patients.

The final domain of ASD surgery that has seen significant advancement in its use of predictive analytics has been regarding HRQOL outcomes for ASD patients following surgical correction [54–56]. This is a vital component of the use of predictive analytics, as patients commonly seek to better understand how surgical interventions will tangibly affect their quality of life. Oh et al. were among the first groups to consider this aspect of postoperative outcomes, and through the use of an ensemble of bootstrapped decision trees developed a predictive model with an accuracy of 85.5% and AUC of 0.96 to determine rates of achieving minimum clinically important difference (MCID) in their 2-year ODI scores [54]. Patients who were predicted to meet the ODI MCID also had significantly higher quality adjusted life years (QALY) gained at 2-year follow-up. Of note, radiographic parameters were not shown to be highly predictive in this model, with top predictors including patient comorbidities (preoperative depression, arthritis, and osteoporosis) as well as number of levels fused. Scheer et al. followed-up this study by considering only patients with preoperative ODI > 30, and built a similar predictive model with 86% accuracy and AUC of 0.94 incorporating 198 patients in their study. An interesting result of these two comparative studies was that when the preoperative baseline ODI score was changed from 15 to 30, the final model identified different variables as the most significant predictors of MCID at 2-years, showcasing the utility of supervised machine learning methods. Major predictors of positive outcomes in patients with a preoperative ODI > 30 included gender, lower preoperative SRS-22 scores, back pain rating and radiographic parameters such as SVA and pelvic incidence to lumbar lordosis (PI-LL) mismatch. Giving surgeons the ability to better predict QOL impacts for patients based on their specific presentations and medical histories will lead to better-informed patient selection and surgical planning, in turn maximizing both patient benefits and resource utilization.

### Advanced uses of machine learning for ASD

While each of the studies described above represents significant forays into the wide-spread adoption of predictive analytics for ASD surgery, there are still improvements to be made in both methodology and applicability. Many of the early predictive models use relatively simple machine learning methods like decision trees, which can have a high propensity for overfitting their models. In addition, a large portion of these studies are limited by their sample size, which is a larger problem that exists within spine surgery. The careful maintenance and construction of robust databases is resource intensive and mandates collaboration across diverse institutions and spine centers to achieve greater sample sizes. In medicine, we are often presented with class imbalance problems when trying to develop predictive models using machine learning. Class imbalance is a phenomenon common in medical datasets, where one class or outcome can represent the majority of data, while a different class or outcome represents a significant minority. As a result of this disparity, predictive models trained on imbalanced data can be heavily biased towards the majority outcome, providing high AUC, accuracy and sensitivity, but unemployable sensitivity when predicting outcomes/events with low incidence. Techniques such as cost-sensitive learning, employment of alternative (more complex) algorithms, and under/oversampling the majority and minority classes respectively, can help mitigate possible imbalance. Taking these shortcomings into consideration, we will next explore a few landmark studies that utilized additional, higher quality methodologies and datasets to create even more robust predictive analytics.

Significant efforts have been undertaken by the ISSG and ESSG in publishing pioneering studies in the field of predictive analytics for ASD surgery. Through an immense collaborative venture spanning multiple countries, spine centers, and numerous surgeons, the ISSG and ESSG have curated a high-quality (as described earlier) and comprehensive database of ASD patients through which they have developed groundbreaking complex analytics. To substantiate the earlier pilot studies demonstrating the feasibility of using predictive models for HRQOL metrics, Ames et al. published what is currently the most expansive study on predicting patient reported outcomes (PROs) [56]. In their model 570 ASD prospectively collected ASD patients were surveyed to assess the probability of achieving MCID in the three major domains of HRQOL metrics for spine surgery: ODI, SRS-22, and SF-36 scores at one- and two-year follow-up. This comprehensive study encompassed 75 variables as input features for model development, and assessed the performance of eight different machine learning algorithms to determine optimal prediction of MCID in the three HRQOL scores. Each algorithm was trained at four distinct time horizons: preoperative baseline, during the immediate postoperative period, at one-year follow-up, and at two-year follow-up. Model performance was assessed using mean absolute error (MAE) as opposed to accuracy and AUC used in earlier predictive models, and final model selection was based on minimization of MAE as well as goodness of fit using R^2^. MAE values across the selected models ranged from 8 to 15%, indicating successful model fitting and highly accurate predictive capabilities [56]. A significant finding from this study was that baseline PROs were the most important variables for predicting final PRO values, while age was the most important objective, patient-level variable, followed by patient comorbidities. This study was then developed further, in an attempt to use machine learning models to predict patient responses to each individual question in the SRS-22 survey. Through the use of six different machine learning algorithms and 150 total patient variables as input features, Ames et al. were able to successfully build a model predicting individual patient answers to each of the SRS-22 questions, with AUC ranging from 0.57 to 0.87 [57]. The significance of this study lies in the level of granularity the authors were able to achieve with their predictive model. The models most accurately predicted patient responses to SRS-22 questions pertaining to the domains of pain, disability and social and labor function, and were less sensitive to predicting responses to questions regarding general satisfaction, appearance, and depression/anxiety. In being able to predict MCID at one- and two-year follow-up, as well as individual patient responses to the SRS-22 survey questions, the authors are pushing ASD management into the era of individualized and personalized medicine that has revolutionized other fields of medicine such as cancer therapy. By leveraging advanced computational techniques, ASD surgeons are now able to substantiate their clinical recommendations with novel and robust data that can tailor decision making and treatment regimens to a patient’s specific needs and care goals.

**Fig. 3:**
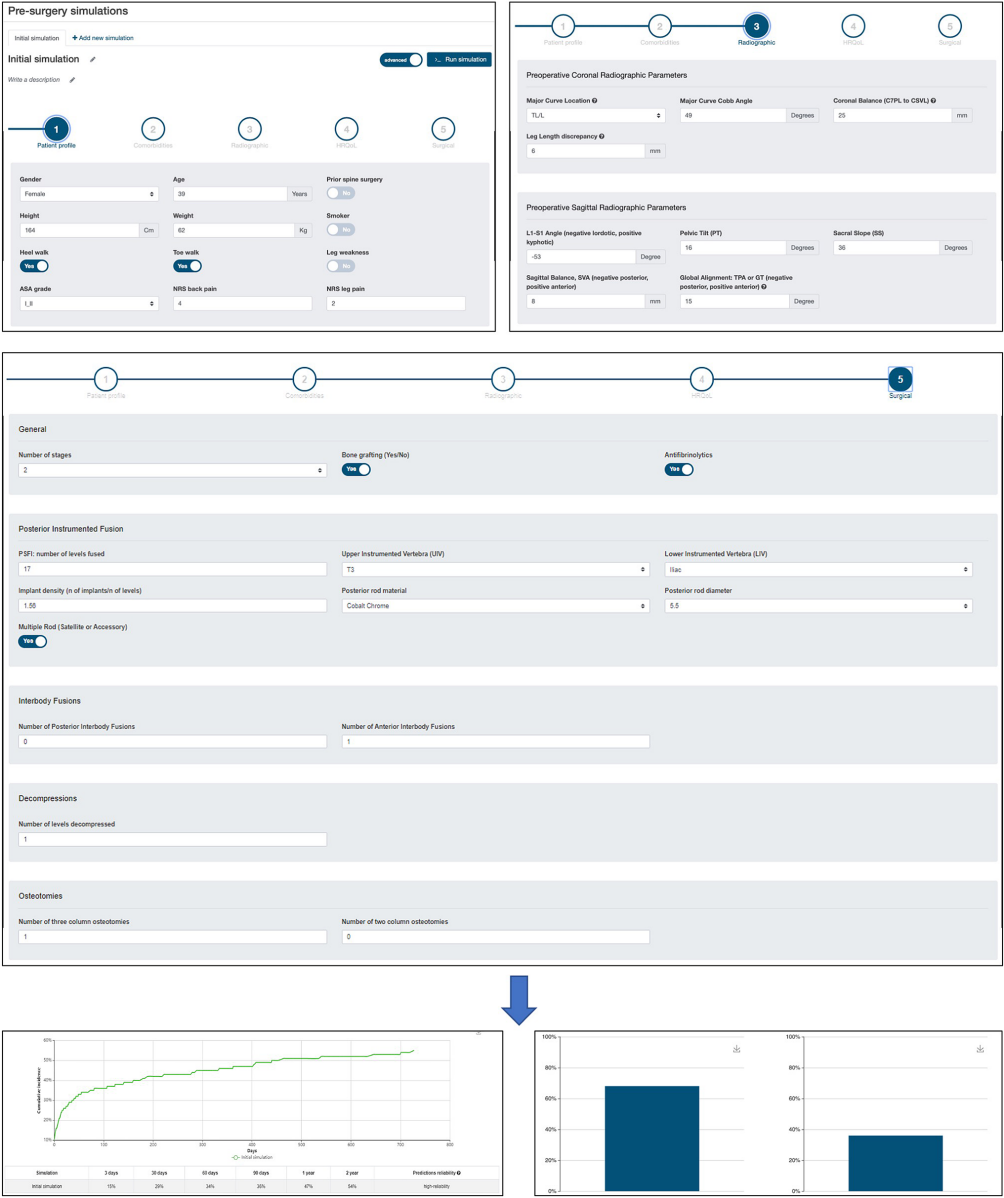
Implementation of a web-based risk calculator for ASD surgery predictions. Patient-related information including demographics (top, left), spinopelvic parameters (top, right) and surgical planning (middle) can be input to run a simulation and predict rates of major complications (bottom, left) and HRQOL outcomes (bottom, right) for the patient

Building upon the earlier predictive analytics for postoperative outcomes and complication risk following ASD surgery, the ISSG and ESSG similarly developed rigorous and more technically complex predictive models using their expansive ASD datasets to enhance predictive capabilities [58]. The relatively high complication rates associated with ASD surgery remain a palpable concern for patients when considering surgical management of their condition. As such, it is crucial that surgeons continue refining predictive models in an effort to provide patients with the most accurate estimates and predictions regarding their outcomes after surgical intervention. These recommendations are currently made based on surgeons’ personal experience and decades of clinical judgment; however, the implementation of predictive models can help elucidate additional information for patients and capture the subtleties and complexities of ASD. Thus, to model major complications (MC), hospital readmission (RA) and unplanned reoperation (RO) rates in patients seeking surgical treatment for ASD, Pellise et al. utilized random forest models encompassing 105 clinical and radiographic variables in an impressive cohort of 1612 prospectively collected ASD patients for model generation. This study was unique in that two models were designed for each of the three outcomes, with the first using all available preoperative information, and the second with the same information in addition to immediate postoperative outcomes (EBL, operative time, surgical procedure, etc..). Using standard training/testing principles, their study achieved adequate predictive accuracy with AUC ranging from 0.67 to 0.92 across the various predictive models[58]. In the MC models, LIV (specifically extension to pelvis) was one of the most important predictors, as were age, walking ability and sagittal deformity radiographic parameters. For RA, pelvic tilt, LIV, age and ODI walking response accounted for the majority of overall predictive power, and notably site and surgeon accounted for a larger portion of predictive power compared to the MC models. In the RO models walking ability was the strongest predictor identified, while site and surgeon accounted for larger predictive power than in both the MC and RA models. The predictive analytics described in this study can prove immensely useful to spine surgeons in many aspects, including surgical candidate selection, and resource optimization by minimizing complication and readmission risks in patients. In an effort to make this information more readily accessible when counseling patients, the ISSG/ESSG have developed a web-based calculator to simulate surgical outcomes and risk profiles for patients based on their specific demographic, radiographic, medical and surgical information (Fig. 3). Online tools such as this calculator can help facilitate the wide-spread adoption of predictive models in the clinical setting, augment surgeon decision making by simulating surgical interventions and their corresponding outcomes (major complication, readmission and reintervention rates, as well as HRQOL outcomes), and allow surgeons to compare simulated outcomes and risk profiles for different surgical strategies.

Moving beyond predicting complications and outcomes, Ames et al. additionally applied similar methodology to predict patients who may experience catastrophic costs following surgical correction of ASD at 90-day and 2-year time points to better understand the economic impact of ASD surgery [59]. Through the use of random forest models and regression trees, models achieved goodness of fit *R*^2^ measures ranging from 56 to 57% for 90-day direct cost, and 29–35% for 2-year direct cost prediction. In addition, the generalized linear regression models used by the authors with forward stepwise selection were able to explain 81% and 64% of the variance in direct cost at 90-days and 2-years, respectively. While these metrics may reflect relatively lower predictive accuracies compared to other simpler models, their design allowed for easier interpretation of model results, and importantly the authors were able to identify variables such as number of levels fused, surgical approach, use of interbody fusion, length of hospital stay, and the attending surgeon as the top predictors of both direct cost, and catastrophic cost as well. The identification of patients who may be at risk of incurring catastrophic costs following ASD surgery may help healthcare initiatives to bundle payments for high-impact and resource intensive treatments like ASD surgery, as well as provide surgeons and hospitals with insight into means of cost reduction in ASD surgery.

Lastly, in the most advanced use of machine learning and AI for ASD surgery to date, Ames et al. published for the first time the use of powerful, unsupervised learning algorithms to develop a novel classification system for ASD patients [60]. In this case, a different approach than previously described models was undertaken by the authors. Unsupervised learning occurs when the data that is being modeled is not “labeled”, or have a direct output defined by the users—this is in direct contrast to earlier supervised learning methods where all of the historical data used to train the predictive models was labeled with the desired output, such that the model could then generate predictions for the specified outcome. The power of unsupervised learning lies in its ability to freely investigate the data for patterns that may intrinsically exist between variables present in the data. Since no particular outcome is specified by the user, the model is free to model the natural structure of the available data. In this case, 570 prospectively collected patients with baseline, one year and two-year follow-up data were included in the study. Harnessing an algorithm known as hierarchical clustering, the authors sought to identify different clusters of ASD patients to better classify patients based on a comprehensive set of input features (patient and surgical characteristics, PRO data, and demographic information), rather than simply radiographic features which had been the gold standard up until that point. This analysis revealed three discrete ASD patient types (patient cohorts) based on their collective characteristic profiles: young patients with coronal deformity, older patients with high incidence of prior spine surgery, and older patients with low incidence of spine surgery. Each of these clusters was unique and exhibited distinct complication and outcomes profiles. When clustering was conducted based on solely surgical characteristics, four unique cohorts of ASD patient types (surgical cohorts) were identified: patients with high number of levels fused and 3CO osteotomy use, patients with high number of levels fused and SPO usage, patients with no osteotomy or interbody fusion, and patients with the highest use of interbody fusion. The generation of three distinct patient cohorts and four distinct surgical cohorts allowed the authors to generate an efficiency grid based on the 12-sub-group intersection of the patient and surgery cohorts, to conduct a risk–benefit analysis. The purpose of the efficiency grid was to delineate mean 2-year PRO and major complication rates for each of the 12 subgroups, highlighting the hypothetical safety and potential outcomes (risk-to-benefit) following any of the four surgical approaches in each of the three homogenous patient cohorts. By comparing the risk-benefits of surgical interventions/approaches over homogenous patient groups using the efficiency grid, spine surgeons will be able to conduct more informed hypothesis testing rather than it necessarily being used for causal inference. For example, the efficiency grid showed across the nine different outcome variables that patients from the “old revision” cohort (elderly patients with higher incidence of prior surgery) undergoing surgery that included 3CO (“3CO” surgical cluster) face considerably higher risk of complications than patients treated without an osteotomy or interbody fusion (“No osteotomy/No interbody fusion” surgical cluster), however the “3CO” surgical cluster patients in the “old revision” cohort experienced overall greater improvements in PROMs. This level of granularity and ability for direct comparisons across surgical intervention and/or patient population once again emphasizes the immense potential of machine learning algorithms to individualize treatment plans for patients based on their unique presentations and histories.

## Proof of concept: novel applications of machine learning for ASD

### Benchmarking to set performance standards

**Fig. 4:**
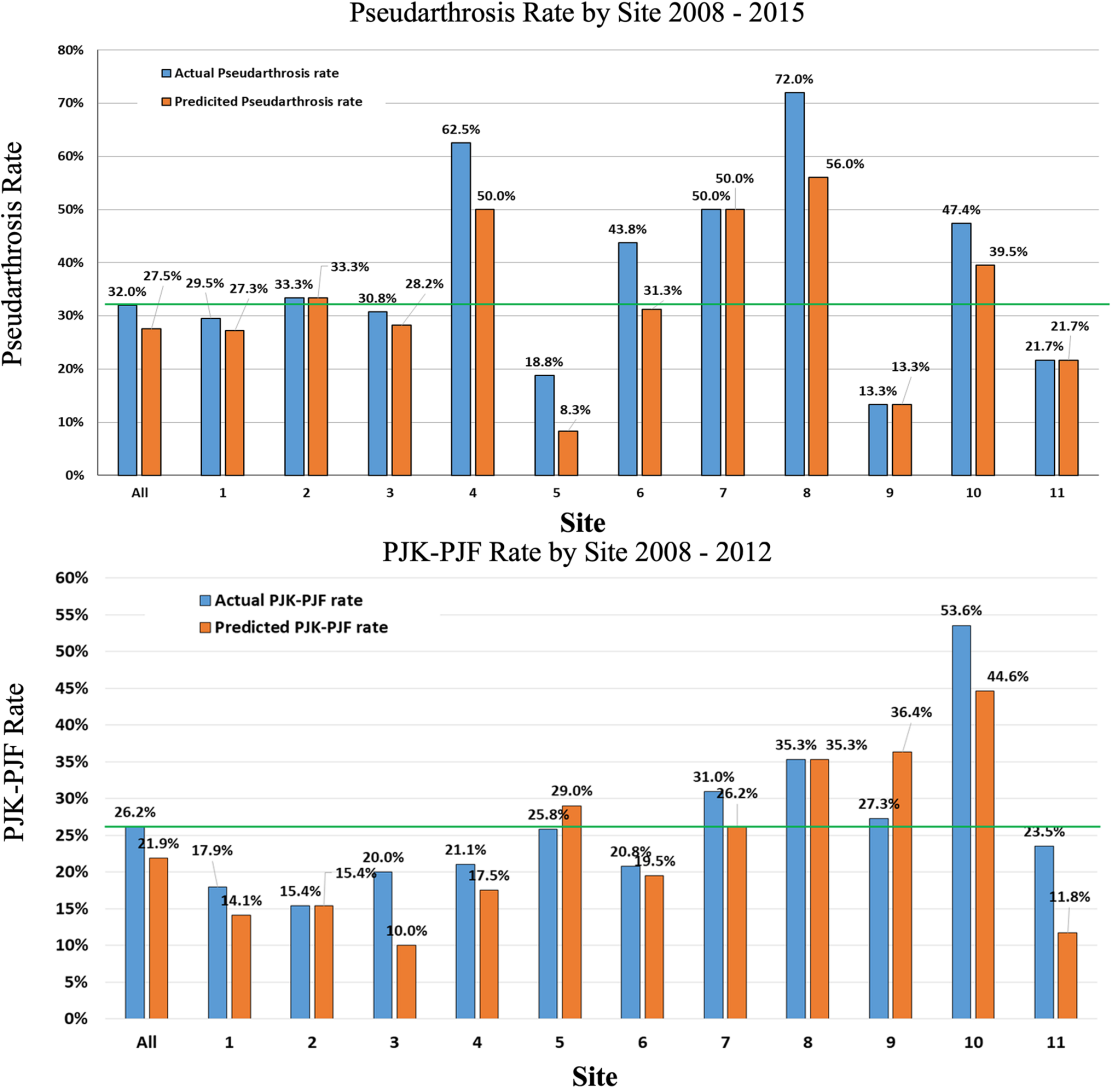
Using predictive modeling for pseudarthrosis and PJF-PJK performance benchmarking in patients with minimum 2-year follow-up. Actual rates for pseudarthrosis and PJF-PJK are compared to site-specific predicted rates. The green line indicates average overall complication rate across all sites. Even though sites may have rates higher than the overall average, it is important to consider their higher predicted rates as well. Several sites demonstrated actual rates below the overall average but were still shown to be underperforming based on their predicted rates, while a couple showed actual rates higher than the average but lower than their predicted rates indicating higher performance

Now that the groundwork has been laid for developing predictive analytics in ASD surgery, it is important to consider more diverse applications of machine learning techniques to push the field further. The computational power encapsulated by these algorithms can provide remarkable insight into numerous facets of ASD surgery. One such application is the use of predictive models for establishing performance benchmarking in spine centers. Benchmarking is critical to the continual refinement of the ASD surgical treatment plan, as it allows institutions to assess their ability to effectively treat a disease, and subsequently identify areas of improvement. Previously, benchmarking was conducted by assessing rates of various outcomes across many different sites and institutions, and then determining an average rate across this extremely diverse cohort. This however is not an accurate assessment, as there are many nuanced factors that can contribute to the performance of an institution, and as such complication rates and outcomes can vary significantly across centers. Some of these differences could relate to case volume and surgeon experience, patient complexity, as well as institutional support staff and operating room protocols, among many others. To remedy this heterogeneity, the ISSG conducted a pilot study using previously published predictive models for pseudarthrosis [51] and PFJ/PJK [49] rates at 2-year follow-up to determine site-specific rate predictions. Once individual rates of pseudarthrosis and PJF/PJK were predicted, the actual rates at each of these sites were compared to their site-specific predicted rates, rather than the overall average rate across all sites to assess their performance (Fig. 4) [61]. For pseudarthrosis, all of the sites used in the study exhibited actual rates (13.3–72.0%) that were greater than their predicted rates (8.3–56%), except for four centers which had the same actual and predicted rates (Fig. 4). Importantly, several sites that had higher actual rates of pseudarthrosis than the overall average, also had higher rates of predicted pseudarthrosis, indicating that different conditions such as patient demographic and/or procedure type may be impacting rates of pseudarthrosis. For PJF/PJK, the majority of sites had lower predicted rates (10.0–44.6%) compared to their actual rates (15.4–53.6%), with the exception of one site that had the same rates, and two sites with lower actual rates (Fig. 4). Notably, sites with actual rates below the overall average were once again still underperforming based on their site-specific predicted rates, and one site that had a higher actual rate than the average was actually performing better than its predicted rate. This preliminary work demonstrates the importance of creating customized predictions for sites based on their respective institutional practices, patients, and other variables for more accurate performance benchmarking. It is important to understand that the predicted rates are not intended to validate or invalidate the models, but rather give more accurate forecasting of what PJK/PJF and pseudarthrosis rates should be based on site-specific and internally acquired data. The disparities seen between actual and predicted rates are minimal for most sites and can likely be explained by variance within our model. However, these differences do indicate a further need to study drivers of these complications and better understand their pathophysiology so surgeons can focus their efforts on prevention.

### Population health studies

**Fig. 5:**
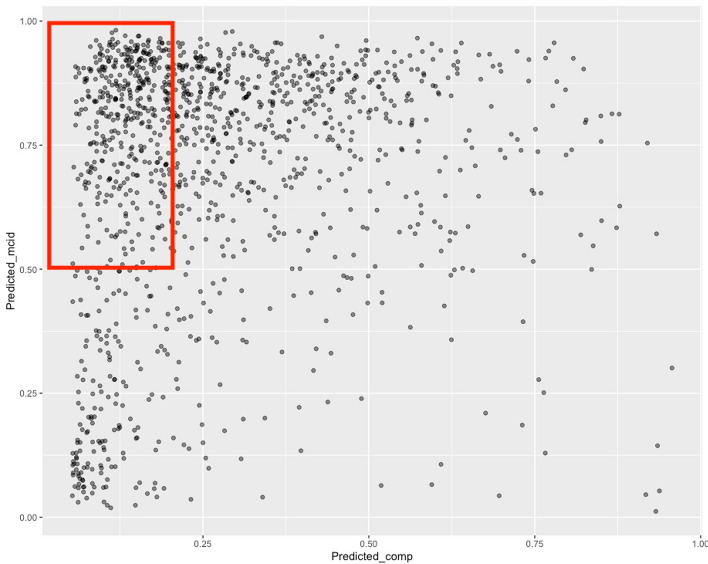
Using a predictive model to simulate patient MCID and complication rates. A total of 1245 patients were simulated using gradient boosting classification, to determine predicted MCID and complication rates. Incremental thresholds were then applied at 10% intervals, to identify sub-populations of patients meeting specific outcomes criteria. The region highlighted by the red box represents patients with predicted MCID > 50%, and complication rate < 20%. This group corresponded to 33% of the original cohort and using these criteria, cost reduction information and decreased surgical utilization rates were extrapolated for ASD surgery in the United States and in Spain

In addition to its performance benchmarking efforts, the ISSG has also explored the utility of applying predictive models for conducting ASD studies at the population level. The motivation behind this study was to better understand how surgical utilization for ASD could be better optimized given the enormous financial burden associated with ASD surgical management. The healthcare costs of first-world economies are accelerating at an unsustainable rate, and better-informed patient selection can help with resource allocation to maximize patient benefits while also relieving some of the associated direct costs of complex disease treatment. To perform this study, the ISSG used previously established predictive analytics for preoperatively determining rates of MCID and complication risk for patients to assess their feasibility for simulating population-level health data. A total of 1245 prospectively collected patients treated at 17 different ASD centers were pooled for this analysis, and clinical outcomes of MCID, complication, and reoperation rates were predicted using gradient boosting classification. Patients were then stratified in increments of 10%, based on their predicted MCID and complication rates. Creating increments of 10% in both of our outcome variables allowed us to comprehensively profile the risk-to-benefit of surgery, and understand the financial implications at each of the thresholds. To determine a cohort of optimal surgical candidates, we considered the sub-group of patients with predicted MCID rates > 50% and complication rate < 20% as a hypothetical simulation, to model the population likely to benefit most from surgical correction (Fig. 5) [62]. These selection criteria corresponded to 33% of the patients in the prospective database being deemed viable candidates for surgery. When these proportions were extrapolated to public health data from both the United States (US) and Spain, significant cost savings were observed. In the US, using $120,000 as the average hospital cost for ASD intervention, the application of our simulated patient criteria would have translated to overall hospital savings of $541 million in the year 2013. In Spain, a similar decrease in surgical utilization was observed, with surgery rate per 100,000 adults dropping from 1.64, to 0.54 based on year 2015 data. The enormous cost reduction observed in this study showed that accurate prognostic models can be used to guide clinical decision making by preoperatively identifying patients who would benefit most from surgery, prior to incurring the expense of their intervention. By comprehensively profiling incremental thresholds of both clinical benefit as well as surgical risk of complication, predictive models such as this can provide both patients and surgeons with tangible data when deciding on a treatment plan, and whether that includes surgery or not. With the large number of ASD surgeries being conducted around the world, better candidate selection may also reduce societal costs and maximize postoperative outcomes in patients, by limiting surgeries with minimal predicted clinical benefits and high complication rates.

## Conclusions

Predictive models and the implementation of machine learning techniques to augment surgeon decision making have substantial potential to improve surgical outcomes for patients with ASD. While significant progress has been undertaken by spine surgeons, there remain several challenges and important obstacles that must be overcome before the wide-spread adoption of predictive analytics in spine practices. The most successful predictive models have been created in the context of a deep understanding of the problem being studied and the underlying data-generating process. As such, it will be imperative moving forward that surgeons across institutions and even across countries collaborate to generate large, comprehensive, and robust databases such that machine learning techniques can be properly utilized. Additionally, there will be a need for consolidation of the various published models, such that surgeons can begin to integrate them into their practices. We hope to see the wide-spread availability and adoption of consolidated calculators and predictive models in the near future, as the ASD calculator developed by the senior authors is currently undergoing alpha testing at various ISSG/ESSG sites. While extremely powerful, great care must also be taken during model development and analytics must be meticulously built using the best practices of machine learning theory. Predictive models only offer partial solutions and must continue to be complemented with rigorous hypothesis testing to truly understand the causal effects in surgery, with further prospective validation for assessment of model performance. This task is certainly complex; however, the challenge ahead should not dissuade surgeons and researchers from continuing to generate predictive and explanatory models. The ability to leverage powerful computational techniques will propel spine surgeons confidently into the era of personalized medicine, allowing them to complement decades of clinical experience and practice with predictive models and insightful data to meaningfully enhance patient care.

## Data Availability

Not applicable.
